# Match Injuries in the Spanish Rugby Union Division de Honor

**DOI:** 10.3390/ijerph191911861

**Published:** 2022-09-20

**Authors:** Roberto Murias-Lozano, Francisco Javier San Sebastián-Obregón, Henar Lucio-Mejías, José Carlos Saló-Cuenca, Gustavo Plaza-Manzano, Ibai López-de-Uralde-Villanueva, José Luis Maté-Muñoz, Pablo García-Fernández

**Affiliations:** 1 Department of Physiotherapy, Camilo José Cela University, Villafranca del Castillo, 28692 Madrid, Spain; 2 Spanish Rugby Federation, 28008 Madrid, Spain; 3 Physioactive Clinic, 28002 Madrid, Spain; 4 Traumatology Unit, Orthopaedic Surgery and Traumatology Service, University Hospital Arnau de Vilanova, 25198 Lleida, Spain; 5 Department of Radiology, Rehabilitation and Physiotherapy, Complutense University of Madrid, 28040 Madrid, Spain; 6 Grupo InPhysio, Instituto de Investigación Sanitaria del Hospital Clínico San Carlos (IdISSC), 28040 Madrid, Spain

**Keywords:** epidemiology, rugby, injuries, lower limb, muscle tear

## Abstract

Objective: To describe the injury rate, severity, cause, anatomical location (tissue damaged), recurrence, place and time during matches throughout a season in the Spanish Rugby Union Division de Honor. Methods: Observational, prospective and descriptive study conducted in the competition of the Spanish División de Honor de Rugby with 258 players. The data were reported by the medical services of the previously formed clubs. Results: Total exposure was 4100 h, during which 220 injuries occurred. The average number of sick days was 36.8. The total injury rate was 53.6 injuries/1000 h of exposure. Three quarters suffered 93 injuries and the forwards sustained a total of 127 injuries, with a total of 48.6 and 58.1 injuries/1000 h of exposure, respectively. Moderate injuries were the most frequent. Specifically, ligament injury was the most frequent, and dislocation was the injury that caused the most sick days. The most injuries occurred in the third quarter of the match, and the most serious injuries occurred in the second quarter. Conclusions: The injury rate of Spanish rugby competitors is 53.6 injuries/1000 match hours, with an average of 36.8 sick days. Contact injuries are the most frequent, taking place especially when tackling or being tackled.

## 1. Introduction

In 1995, rugby was professionalised, which increased the visibility of this sport worldwide. It presents different modalities depending on the number of players in the match (rugby 15, rugby 13 and rugby 7) [1]. Over 12 million people play rugby in the world, both federated and non-federated, in different categories and with great acceptance. There are many followers and high competitive levels in countries such as England, France, Wales, Ireland, Scotland, Italy, South Africa and Australia [1].

The most common injuries in rugby usually occur from direct contact between players [2,3,4,5,6,7,8]. For its part, tackling is the most injurious element of rugby, although other elements such as the maul, the melee, the scrum and the ruck are also injury risk factors [2,3,4,5,6,7,8].

Moreover, it is important to highlight that the most injuries, and the most serious ones, occur during the matches, increasing exponentially with age and game level [2,8,9,10,11]; moreover, the number of injuries is strongly influenced by the competitive pressure that players are subjected to, in addition to the lack of rest between competitions, since it has been established that this lack of rest significantly increases the risk of suffering an injury [10].

The injury rates in the 2007, 2011 and 2015 World Cups ranged between 83.9 and 90.1 injuries/1000 competition hours [4,5,7]. In women, this rate was 37.5 injuries/1000 h of competition in the 2006 World Cup and 35.5 injuries/1000 h in the 2010 World Cup [12,13]. This high injury rate leads to a large number of sick leave days, according to several authors between 18 and 37.4 days, implying a high economic and personal cost [3,8,14]. Other sports such as football present a significantly lower rate (27.5 injuries/1000 h) [15].

In rugby, considering that it is a sport where different physical complexions are typified, there are differences in the type of injuries depending on the position occupied by the player: Forwards present more injuries due to physical contact, such as fractures or ligament injuries and dislocations, whereas the three-quarters accumulate more muscular injuries due to a greater explosiveness in their game, mechanically stressing the muscles involved [16].

Currently, epidemiological studies allow for observing and analysing injuries in high-performance athletes, which can help to prevent such injuries [17]. This is fundamental in contact sports like rugby, where serious injuries take place such as concussion, which is very frequent in matches compared with training sessions [18].

To the best of our knowledge, no study has exclusively analysed match injuries in the Spanish División de Honor. Therefore, the aim of this study was to describe the injuries and the variables that influence these injuries during a rugby match, distinguishing the results by playing position (rate, anatomical location, tissue, type, seriousness, lesional mechanism, cause, place, time of the match, recurrence) throughout a full season.

## 2. Material and Methods

### 2.1. Study Design

This study used an observational, prospective and descriptive design, and it was approved by the Ethics Committee of the Camilo José Cela University of Madrid.

### 2.2. Participants

All the teams in the Spanish División de Honor de Rugby were invited to participate in the study. Each club was informed of the methodology and objectives of the study, as well as about the steps to follow and the terminological consensus required to record the injuries. Such data were treated according to Organic Law 3/2018, of December 5th, on the Protection of Personal Data and guarantee of digital rights.

Only the injuries that took place in matches throughout a full season were recorded, including those sustained in pre-season matches. The total sample was constituted by 258 players who were federated in the study season. 

### 2.3. Data Gathering

The data gathering was carried out once per week by the medical services of the eight clubs participating in the study. This data gathering was always conducted by the same professional in order to minimise possible biases in the diagnosis of the injuries. For the recording of the data, these professionals used a data-gathering sheet based on the consensus proposition of Fuller et al. [19] for the realisation of epidemiological studies, adding some relevant questions for the analysis of injuries, employing the nomenclature of Fuller et al. [19] at all times. Before initiating the study, the players were informed of the nature and characteristics of the study, and they signed an informed consent document. The study was performed in compliance with the principles of the Declaration of Helsinki for research on human beings [20] and was approved by Ethics Committee of Camilo José Cela University of Madrid.

### 2.4. Inclusion and Exclusion Criteria

All the players with a contract in the first team participated in the study. The players with previous injuries and those who presented injuries at the time of initiating the data gathering were not excluded, although their injuries were not included in the study for statistical reasons, and the exposure factor was not considered until the player had recovered from such injury. Only those injuries sustained during matches were included. The players who finished the season injured or left the club during the season while injured were followed up until their ultimate discharge date. 

### 2.5. Definition of Injury

Following the consensus proposition of Fuller et al. [19], injuries were considered “any physical complaints suffered by a player during a match or training session that prevented the player from fully participating in all the training activities or matches for more than one day after the injury, regardless of whether matches or training sessions were scheduled”. Recurring injuries were “those that coincided in type and anatomical location with the previous injury, occurring after returning to full participation once recovered from such previous injury”. Anthropometric data (weight, height), body dominance, type of injury, location, tissue and seriousness were recorded.

Injury rate was analysed as the number of injuries per 1000 h of match exposure. Exposure was calculated for 80 min for 15 players (8 forwards and 7 three-quarters) in every match and on every team. Injury seriousness was categorised as minimal (2–3 days), mild (4–7 days), moderate (8–28 days), serious (>28 days) and sports withdrawal [17].

### 2.6. Statistical Analysis

The statistical analysis was conducted using SPSS v.25 software for Windows (IBM SPSS: Statistical Package for Social Science, Chicago, IL, USA). The analysis of the variables was expressed in means and standard deviations (SD) and their 95% confidence intervals (CI). The normality of the data was analysed using the Kolmogorov–Smirnov test. The differences of means were analysed with parametric or non-parametric tests depending on the data normality results. The correlations between variables were analysed with Pearson’s and Spearman’s correlation coefficients. The associations between qualitative variables were analysed with chi-squared comparisons. The significance level was set at *p* < 0.05.

## 3. Results

### 3.1. Anthropometric Characteristics and Sports Habits 

The final study sample consisted of 258 professional players (three-quarters: 114; forwards: 144) of the Spanish División de Honor de Rugby. The mean age of the players was 25.4 ± 4.6 years, the mean height was 181.6 ± 7.2 cm and the mean weight was 94.0 ± 14.1 kg (Table 1). Statistically significant differences between the player positions were found in height, weight (*p* < 0.001) and age (*p* = 0.005) but not in years of experience (*p* = 0.306), with the forwards being taller, heavier and older, on average, than the three-quarters. 

Total exposure was 4100 h (three-quarters: 1913; forwards: 2187) during the 205 matches played, with a total record of 220 injuries (three-quarters: 93; forwards: 127).

The injury rate in matches was 53.6 injuries/1000 h of match exposure (95% CI: 46.7–60.5). By positions, the three-quarters presented 48.6 injuries/1000 h (95% CI: 38.9–58.2), whereas the forwards presented 58.1 injuries/1000 h (95% CI: 48.2–67.8). 

### 3.2. Injury Seriousness

The 220 injuries resulted in a total of 8321 sick days, with an average of 36.8 (95% CI: 30.1–43.6). The forwards presented a greater injury rate 127 injuries (57.73%) 58.0/1000 h (95% CI: 48.2–67.8) than the three-quarters 93 injuries (42.27%) 48.6/1000 h (95% CI: 38.9–58.25). However, the injuries represented fewer sick days in the forwards (32.28 (95% CI: 20.35–40.2)) than in the three-quarters (43.1 (95% CI: 31.3–54.9)). Moderate injuries were the most frequent (82 injuries; 37.2%), with an injury rate of 20.0/1000 h (95% CI: 15.7–24.2), which represented an average number of sick days of 16.3 (95% CI: 14.9–17.6), followed very closely by serious injuries (81 injuries; 36.8%), with an injury rate of 19.7/1000 h (95% CI: 15.5–24.0) and an average number of sick days of 80.3 (95% CI: 66.4–94.2). By positions, the forwards suffered more moderate injuries (55 injuries; 25.0%) (16.7 sick days; 95% CI: 14.9–17.6) and the three-quarters recorded more serious injuries (42 injuries; 19.0%) (83.00 sick days; 95% CI: 62.4–103.5), without statistically significant differences in the sick days between positions (*p* = 0.142). The injury rate and number of sick days as a function of seriousness is shown in Table 2.

### 3.3. Anatomical Location

The most frequent general anatomical location of the lesion was the lower limb with 114 lesions (51.8%) (27.8 injuries/1000 h.; 95% CI: 22.7–32.8) followed by the upper limb (50 injuries; 22.7%) (12.2 injuries/1000 h.; 95% CI: 8.8–15.5). By positions, the forwards presented 72 injuries in the lower limb (56.7%), whereas the three-quarters presented 42 injuries (45.2%). The specific anatomical location with the greatest injury rate was the head/face (8.5 injuries/1000 h.; 95% CI: 5.7–11.3), followed by the ankle (8.2 injuries/1000 h.; 95% CI: 5.5–11.0) and the knee (7.5 injuries/1000 h.; 95% CI: 4.9–10.2). Regarding seriousness, the upper limb injuries showed the most sick days (51.0 days; 95% CI: 31.9–70.0), followed by the lower limb (37.7 days; 95% CI: 28.6–46.8). The specific anatomical location with greatest seriousness was the knee (74.7 days; 95% CI: 48.8–100.5), followed by the shoulder/collar bone (64.6 days; 95% CI: 29.5–99.7) and the arm (60.8 days; 95% CI: −21.3–143.9) (Table 3). Statistically significant differences were identified in sick days as a function of the anatomical location (*p* = 0.005), with the upper limb injuries causing the most sick days.

### 3.4. Injured Tissue

Joint injury was the most frequent (88 injuries; 40.0%) (21.4 injuries/1000 h; 95% CI: 17.0–25.), and it also caused the largest average number of sick days (50.5 days; 95% CI: 36.3–64.8). After joint injury, the second most frequent injury was muscle injury (51 injuries; 23.2%) (12.4 injuries/1000 h; 95% CI: 9.0–15.8), causing an average number of 23.96 sick days (95% CI: 15.7–32.2). By positions, both in forwards and three-quarters, the most frequent injury was also joint injury, with 50 injuries (22.3%), 22.8 injuries/1000 h (95% CI: 16.6–29.1) and 40.3 sick days (95% CI: 25.9–60.7) in the forwards, and 38 injuries (17.2%), 19.86 injuries/1000 h (95% CI: 13.6–26.1) and 60.1 sick days (95% CI: 35.7–84.4) in the three-quarters, although the sick days of the joint injuries were more serious in the three-quarters than in the forwards (Table 4). 

The sick days showed statistically significant differences according to the injured tissue (*p* < 0.001), with the most sick days being caused by joint injuries (50.5 days; 95% CI: 36.3–64.8), followed by bone injuries (49.5 days; 95% CI: 33.0–66.0). Statistically significant dependences were found between the injured tissue and the seriousness of the injuries (*p* ≤ 0.001); the joint injuries tended to be serious, whereas the skin and muscle injuries tended to be mild. There were no statistically significant differences between the playing positions of the players. Moreover, statistically significant dependence was also observed between the injured tissue and recurrence (*p* = 0.021), with the joint injuries tending to be recurrent and the bone injuries tending to be non-recurrent.

### 3.5. Type of Injury

Ligament injury was the most frequent type (72 injuries; 32.7%) (17.5 injuries/1000 h; 95% CI: 13.5–21.5) (43.4 sick days; 95% CI: 30.0–56.8), both in general and by position. However, regarding the number of sick days, dislocation/subluxation was the most serious type of injury (95.0 sick days; 95% CI: 32.3–157.6), both in general and by position, with 95.7 days (95% CI: 2.4–188.9) in forwards and 94.1 days (95% CI: 28.3–216.6) in three-quarters (Table 5). 

The sick days showed statistically significant differences depending on the type of injury (*p* < 0.001), with dislocations being associated with the most sick days. There were no statistically significant differences between the playing positions of the players.

### 3.6. Lesional Mechanism

The most frequent mechanism of injury by contact was tackling (53 injuries; 24.1%) (12.9 injuries/1000 h; 95% CI: 9.4–16.3), followed by non-contact injuries (47 injuries; 21.4%) (11.4 injuries/1000 h; 95% CI: 8.2–14.7) and being tackled (46 injuries; 20.9%) (11.2 injuries/1000 h (95% CI: 8.0–14.4). By positions, non-contact injuries were the most frequent in forwards, whereas the three-quarters sustained more injuries when tackling. Regarding seriousness, being tackled was the most serious injury (56.8 sick days; 95% CI: 35.8–77.8), followed by the maul (52.5 days; 95% CI: 47.1–262.1) and tackling (40.1 days; 95% CI: 24.3–56.0) (Table 6). Statistically significant differences were observed in the sick days based on the lesional mechanism (*p* < 0.001), with being tackled showing the most serious injuries. There were no statistically significant differences between the playing positions of the players.

### 3.7. Cause of Injury

The most frequent cause of injury was trauma (163 injuries; 74.1%), followed by overuse (57 injuries; 25.9%). Both forwards and three-quarters presented more injuries by trauma, with 87 injuries (68.5%) and 76 injuries (81.7%), respectively. No statistical differences were observed in the sick days based on the cause of injury (*p* = 0.196).

### 3.8. Surface

A total of 160 out of the 220 injuries took place in natural grass fields (72.7%). By positions, both forwards and three-quarters suffered more injuries on natural grass (70.9% and 75.3%, respectively). There were no differences in the sick days between natural and artificial grass (*p* = 0.088).

### 3.9. Time of the Match

The most frequent injuries took place in the third quarter (77 injuries; 35.0%) and in the fourth quarter (62 injuries; 28.2%). The forwards presented greater percentage of injuries during the third quarter (52 injuries; 40.9%), whereas the three-quarters suffered more injuries during the fourth quarter of the match (26 injuries; 28.0%). The greatest rate corresponds to the third quarter (18.7 injuries/1000 h; 95% CI: 14.6–22.9), followed by the fourth quarter (15.1 injuries/1000 h; 95% CI: 11.3–18.8). The forwards presented greater injury rate in the third quarter (23.7 injuries/1000 h; 95% CI: 17.3–30.1), whereas the three-quarters showed greater injury rate in the fourth quarter (13.5 injuries/1000 h; 95% CI: 8.4–18.7). However, the injuries were more serious in the second quarter (52.8 sick days; 95% CI: 31.7–73.8), both in general and by position (forwards: 43.4 sick days; 95% CI: 18.1–68.7) (three-quarters: 63.3 days; 95% CI: 27.0–99.6) (Table 7). Statistically significant differences were observed in the sick days based on the time of the match (*p* < 0.029), with the second quarter concentrating the most serious injuries. There were no statistically significant differences between the playing positions of the players.

### 3.10. Injury Recurrence

Of the total 220 lesions, 24.0% were recurrent. The forwards presented 29 recurrent injuries (13.1% of the total), whereas the three-quarters suffered 24 recurrent injuries (10.9% of the total). Regarding seriousness, the new injuries caused more sick days, both generally and by position (Table 8).

There were no statistically significant differences in sick days either when analysing the whole sample or between the playing positions of the players.

## 4. Discussion

To the best of the authors’ knowledge, this is the first study conducted in Spain to exclusively analyse the injuries caused during matches in the Spanish División de Honor de Rugby, contributing to the knowledge about them with the aim of establishing future prevention strategies.

The mean age, weight and height of the players were 25.4 ± 4.6 years, 94.0 ± 14.1 kg and 181.6 ± 7.2 cm, respectively. They presented statistically significant differences between positions in height, weight (*p* < 0.001) and age (*p* = 0.005), with the forwards being taller, heavier and older, on average, than the three-quarters, and they showed differences in anthropometric values between positions similar to those reported by Whitehouse et al. [8]. However, these differences did not cause a difference in seriousness between positions (*p* = 0.142).

Our study obtained an average number of 36.8 sick days, which is in line with the results of studies such as that of Schwellnus et al. [11], although this finding is considerably different from that of other studies, where the average number of sick days was significantly lower [5,6,7]. This difference could be due to the fact that these studies were carried out during different editions of the Rugby World Cup, which could have meant players received less conservative treatment due to the immediacy of the matches in time and the need for an early return to the competition, as well as to a lower seriousness of the injuries, compared with our study. However, given the large number of sick days recorded in competition in the Spanish league, we consider it necessary to closely monitor the evolution of the injuries in subsequent seasons in order to implement preventive measures that allow for reducing the seriousness of the injuries, thus increasing safety in rugby play.

The injury rate in matches in the Spanish División de Honor was 53.6 injuries/1000 match hours (95% CI: 46.7–60.5). This result is similar to those reported in other studies, in which the injury rate was 52.0–55.8 injuries/1000 match hours [2,3]. Other studies carried out in rugby present higher injury rates, ranging between 66.0 and 138.0 injuries/1000 match hours [5,6,7,8,20,21]. The fact that these studies were conducted in higher leagues and in international tournaments and championships could be the cause of these differences since the physical level of the players who participate in these competitions is higher, and so are the intensity and demand, due to the competitive level of such tournaments. 

By positions, this study recorded an injury rate of 48.6 injuries/1000 match hours (95% CI: 38.9–58.2) for the three-quarters and 58.1 injuries/1000 match hours for the forwards (95% CI: 48.2–67.8). Several studies report greater rates in forwards than in three-quarters [4,11,22], whereas other studies indicate that the position of three-quarters showed greater rates [5,8,23,24,25,26]. Therefore, we cannot clearly identify which position presents the highest risk of injury, although it seems logical to think that the participation of the forwards in play actions with greater contact would make them prone to more injuries.

Despite suffering more injuries, the forwards in this study suffered less serious injuries, which caused 32.3 sick days on average, compared to the average 43.1 sick days of the three-quarters. However, there were no statistically significant differences in the seriousness of the injuries based on the position of the players (*p* = 0.142).

The most frequent anatomical location in this study was the lower limb, both in general and by position. This finding is in line with those reported by other studies carried out in rugby, which present a range of 46.9% to 57.1% of total injuries [2,6,7,8,10]. Nevertheless, the upper limb caused more sick days, both in general and by position. Thus, in spite of a greater injury rate in the lower limb, the injuries that affect the upper limb seem to be more serious. 

The joints sustained 40% of the injuries, followed by the muscle-tendon injuries (23.2%). These data are similar to those of the majority of studies on rugby injuries, which also present greater presence of muscle and joint injuries [2,6,11]. 

The most frequent specific type of injury in our study was sprain (32.7%), followed by muscle rupture, overload or tear (20.9%). These data are in line with those reported in previous studies, where the most common injuries were sprain and the injuries that affect the muscle [2,7,10,21,27]. We consider this finding to be very relevant, given the current concern in rugby about the study of concussions, which could pose lower monitoring of ligament and muscle injuries, thereby increasing the rate of this naturally frequent type of injuries.

Moderate and serious injuries were the most common in our study (37.3% and 36.8%, respectively). Several studies present similar results [3,4,11]. However, other studies carried out in rugby indicate that mild and minimal injuries are the most common in terms of seriousness [2,8,21,27]. These differences could be due to the methodological characteristics of each study. 

In this study, 79.6% of the injuries were caused by direct contact. Studies carried out in rugby such as those of Solis-Mencia et al. [10] and Williams et al. [28] present similar data regarding the lesional mechanism of contact injuries, which seems logical given the playing characteristics of rugby. Tackling posed the most damaging play action in this study, which is in line with the studies of Fuller et al. [4,6] and Schneiders et al. [2], who present similar data of injuries sustained when tackling. However, other studies present more tackling injuries, with percentages ranging between 21.9% and 44.8% [3,5,7,10]. Despite the disparity of data regarding the most damaging move, there is no doubt that tackling and being tackled are present in most of the injuries of professional rugby. 

The third quarter of the match had a higher concentration of injuries than the other quarters, with 35% of all injuries. These data are in line with those of other studies in which injuries were more frequent in the third quarter [3,4,7,8]. However, it is worth highlighting that the most serious injuries were sustained in the first two quarters of the matches. By positions, the forwards reported more injuries in the third quarter of the match, whereas the three-quarters suffered more injuries in the fourth quarter, which is in line with the findings of Fuller et al. [7] in their study conducted in the 2011 Rugby World Cup.

The main strength of the present study is that it is the first study performed in Spain on the injuries sustained during matches in the maximum category of rugby, describing the characteristics, rate and seriousness of the injuries and using the same methodology of previous studies carried out in higher-level competitions. This study contributes to the knowledge on the injuries that take place during rugby competition and can be used to implement preventive measures that increase safety during rugby practice. It also aims to be a first step in the study of rugby injuries in Spain, laying the groundwork for this and other research groups to conduct rigorous epidemiological studies, such as those conducted in other countries with a long tradition in the practice of rugby, to, through the reduction of the incidence and severity of injuries, increase the competitive level in Spanish rugby.

The present study has some limitations that must be pointed out. The main limitation of the study is that although an adequate methodology was used for data recording, since the data were recorded by the different medical services of the clubs, there may be some differences in the classifications of the injuries. Moreover, the players could not be followed up during the concentrations with the respective national teams. 

## 5. Conclusions

Injuries in the matches of the Spanish División de Honor de Rugby are frequent and serious, with 53.6 injuries/1000 match hours and an average of 36.8 sick days. The lower limb is the most frequent anatomical location, although the most serious injuries affect the upper limb. The injuries by direct contact are the most frequent, occurring especially when tackling or being tackled. Injuries are mostly trauma, most frequent in the third quarter, and more serious in the second quarter.

## Figures and Tables

**Table 1:** Anthropometric data and sports habits injury rate.

	Full Sample		Three-Quarters		Forwards		*p*-Value
	n = 258		n = 114		n = 144		
	Mean	S.D.	Mean	S.D.	Mean	S.D.	
Weight (kg)	94.0	14.1	83.2	8.7	102.5	11.5	<0.001 *
Height (cm)	181.6	7.1	178.4	5.8	184.1	7.1	<0.001 *
Age (years)	25.3	4.5	24.3	3.8	26.2	4.9	0.005 *
Years playing rugby	12.4	5.4	12.3	5.1	12.4	5.8	0.306 †
Days of training/week	3.9	0.7	4.0	0.7	3.9	0.7	0.452 †
Hours of training/day	2.3	0.5	2.4	0.5	2.3	0.5	0.673 †
Hours of training in the gym	3.5	1.5	3.5	1.6	3.5	1.5	0.966 †
Hours of training on natural grass	5.0	2.7	5.0	2.7	5.0	2.7	0.832 †
Hours of training on artificial grass	0.9	1.6	0.9	1.6	0.9	1.5	0.569 †
N.° previous injuries	1.6	1.3	1.6	1.2	1.6	1.3	

SD = standard deviations: kg = kilograms, cm = centimeters. * Student’s *t*-test was used. † Wilcoxon-Mann-Whitney test was used.

**Table 2:** Seriousness of the injuries.

Seriousness	Position	n (%)	Rate (95% CI:)	Sick Days (95% CI:)	*p*-Value
Total		220	53.6 (46.7–60.5)	36.8 (30.1–43.6)	
	Forwards	127 (57.7)	58.0 (48.2–67.8)	32.2 (24.3–40.2)	0.142 †
	Three-quarters	93 (42.2)	48.6 (38.9–58.2)	43.1 (31.3–54.99)	
Minimal (2–3 days)		13 (5.9)	3.1 (1.4–4.8)	2.3 (2.0–2.69)	
	Forwards	5 (2.2)	2.2 (0.2–4.2)	2.0 (1.8–2.7)	
	Three-quarters	8 (3.6)	4.1 (1.2–7.0)	2.6 (2.1–3.0)	
Mild (4–7 days)		42 (19.0)	10.2 (7.1–13.3)	5.7 (5.4–6.1)	
	Forwards	27 (12.2)	12.3 (7.7–16.9)	5.5 (5.1–6.0)	
	Three-quarters	15 (6.8)	7.8 (3.8–11.9)	6.1 (5.5–6.7)	
Moderate (8–28 days)		82 (37.2)	20.0 (15.7–24.2)	16.3 (14.9–17.6)	
	Forwards	55 (25.0)	25.1 (18.5–31.7)	16.7 (15.1–18.2)	
	Three-quarters	27 (12.2)	14.1 (8.8–19.4)	15.4 (12.9–18.0)	
Serious (+28 days)		81 (36.8)	19.7 (15.5–24.0)	80.3 (66.4–94.2)	
	Forwards	39 (17.7)	17.8 (12.2–23.3)	77.4 (57.9–96.9)	
	Three-quarters	42 (19.0)	21.9 (15.3–28.5)	83.0 (62.4–103.5)	
Sports withdrawal		2 (0.9)	0.4 (0.1–1.1)		
	Forwards	1 (0.4)	0.4 (0.4–1.3)		
	Three-quarters	1 (0.4)	0.5 (0.5–1.5)		

CI = confidence intervals. † Wilcoxon-Mann-Whitney test was used.

**Table 3:** Rate and seriousness of the match injuries distributed anatomically.

	Forwards (n° Injuries = 127)			Three-Quarters (n° Injuries = 93)			Total Matches (n° Injuries = 220)		
	n (%)	Rate (95% CI:)	Seriousness (95% CI:)	n (%)	Rate (95% CI:)	Seriousness (95% CI:)	n (%)	Rate (95% CI:)	Seriousness (95% CI:)
Head/Neck	21 (16.5)	9.6 (5.5–13.6)	27.0 (6.1–47.8)	20 (21.5)	10.4 (5.9–15.0)	20.3 (11.8–28.8)	41 (18.6)	10.0 (6.9–13.0)	23.7 (12.7–34.7)
Head/Face	18 (14.1)	8.2 (4.4–12.0)	18.3 (10.6–26.1)	17 (13.3)	8.8 (4.6–13.0)	20.8 (11.4–30.2)	35 (15.9)	8.5 (5.7–11.3)	19.6 (13.8–25.3)
Neck/Cervical spine	3 (2.3)	1.3 (0.1–2.9)	78.6 (216.9–374.2)	3 (2.3)	1.5 (0.2–3.3)	17.0 (34.8–68.8)	6 (2.7)	1.4 (0.2–2.6)	47.8 (39.8–135.5)
Upper limb	27 (21.2)	12.3 (7.7–16.9)	49.1 (23.4–74.8)	23 (17.3)	12.0 (7.1–16.9)	53.2 (22.6–83.8)	50 (22.7)	12.2 (8.8–15.5)	51.0 (31.9–70.0)
Shoulder/Collar bone	13 (10.2)	5.9 (2.7–9.1)	55.2 (4.9–105.5)	12 (9.4)	6.2 (2.7–9.8)	74.8 (18.1–131.5)	25 (11.3)	6.1 (3.7–8.4)	64.6 (29.5–99.7)
Arm	3 (2.3)	1.3 (0.1–2.9)	97.6 (52.0–247.8)	2 (1.5)	1.0 (0.4–2.4)	5.5 (0.8–11.8)	5 (2.2)	1.2 (0.1–2.2)	60.8 (21.3–143.9)
Elbow	1 (0.7)	0.4 (0.4–1.3)	6.0	2 (1.5)	1.0 (0.4–2.4)	11.0 (7.5–15.2)	3 (1.3)	0.7 (0.1–1.5)	9.3 (2.1–16.5)
Wrist	3 (2.3)	1.3 (0.1–2.9)	12.0 (26.8–50.8)	2 (1.5)	1.0 (0.4–2.4)	39.5 (37.4–45.4)	5 (2.2)	1.1 (0.1–2.2)	23.0 (2.1–16.5)
Hand/Thumb/Fingers	7 (5.5)	3.2 (0.8–5.5)	39.2 (14.1–64.4)	5 (3.9)	2.6 (0.3–4.9)	42.8 (4.9–90.5)	12 (5.4)	2.9 (1.2–4.5)	40 (21.2–60.2)
Trunk	7 (5.5)	3.2 (0.8–5.5)	15.8 (1.6–33.3)	8 (8.6)	4.1 (1.2–7.0)	22.2 (6.9–37.6)	15 (6.8)	3.6 (1.8–5.5)	19.2 (9.1–29.3)
Rib cage/Ribs/Sternum	2 (1.5)	0.9 (0.3–2.1)	7.0	2 (1.5)	1.0 (0.4–2.4)	23.5 (19.8–24.8)	4 (1.8)	0.9 (0.0–1.9)	15.2 (12.0–42.5)
Lumbar spine	4 (3.1)	1.8 (0.0–3.6)	10.0 (1.2–21.2)	3 (2.3)	1.5 (0.2–3.3)	6.0 (6.9–18.9)	7 (3.1)	1.7 (0.4–2.9)	8.2 (2.5–14.0)
Abdomen	1 (0.7)	0.4 (0.4–1.3)	57.0				1 (0.4)	0.2 (0.2–0.7)	57.0
Pelvis/Sacrum				3 (2.36)	1.57 (0.20–3.34)	37.67 (13.80–61.54)	3 (1.36)	0.73 (0.10–1.56)	37.67 (13.80–61.54)
Lower limb	72 (56.6)	32.9 (25.4–40.4)	29.0 (20.6–37.5)	42 (45.1)	21.96 (15.3–28.5)	52.6 (32.9–72.2)	114 (51.8)	27.8 (22.7–32.8)	37.7 (28.6–46.8)
Hips/Groin	1 (0.7)	0.4 (0.2–1.35)	68.0	3 (2.3)	1.5 (0.2–3.3)	28.3 (50.9–107.5)	4 (1.8)	0.9 (0.0–1.9)	38.2 (13.8–90.3)
Anterior side of the thigh	11 (8.6)	5.0 (2.0–7.9)	11.7 (5.9–17.5)				11 (5.0)	2.6 (1.1–4.2)	11.7 (5.9–17.5)
Posterior side of the thigh	10 (7.8)	4.5 (1.7–7.4)	18.4 (8.4–28.3)	4 (3.1)	2.0 (0.0–4.1)	31.0 (24.4–86.4)	14 (6.3)	3.4 (1.6–5.2)	22.0 (9.7–34.2)
Knee	15 (11.8)	6.8 (3.4–10.3)	59.2 (26.1–92.3)	16 (12.6)	8.3 (4.2–14.4)	89.1 (47.4–130.9)	31 (14.0)	7.5 (4.9–10.2)	74.7 (48.8–100. 5)
Leg/Achilles Heel	6 (4.7)	2.7 (0.5–4.9)	32.1 (1.7–62.6)	5 (3.9)	2.6 (0.3–4.9)	24.2 (6.8–55.2)	11 (5.0)	2.6 (1.1–4.2)	28.5 (10.9–46.1)
Ankle	22 (17.3)	10.0 (5.8–14.2)	23.0 (12.5–33.4)	12 (9.4)	6.2 (2.7–9.8)	35.5 (3.9–67.0)	34 (15.4)	8.2 (5.5–11.0)	27.4 (15.2–39.5)
Foot/Toes	7 (5.5)	3.2 (0.8–5.5)	17.7 (1.1–36.6)	2 (1.5)	1.0 (0.4–2.4)	13.0 (8.6–14.6)	9 (4.0)	2.2 (0.7–3.6)	16.6 (2.6–30.7)

CI = confidence intervals.

**Table 4:** Injury rate and seriousness distributed by injured tissue and position.

**Injured Tissue**	***p*** **-Value**	**Position**	**n (%)**	**Rate (95% CI:)**	**Seriousness** **(95% CI:)**	***p*** **-Value**
Joint	<0.001 *		88 (40.0)	21.4 (17.0–25.9)	50.5 (36.3–64.8)	
		Forwards	50 (22.3)	22.8 (16.6–29.1)	40.3 (25.9–60.7)	0.703 †
		Three-quarters	38 (17.2)	19.8 (13.6–26.1)	60.1 (35.7–84.4)	
Muscle			51 (23.1)	12.4 (9.0–15.8)	23.9 (15.7–32.2)	
		Forwards	34 (15.4)	15.5 (10.3–20.7)	25.4 (14.4–36.3)	0.057 †
		Three-quarters	17 (7.7)	8.8 (4.6–13.0)	21.0 (8.1–33.92)	
Bone			32 (14.5)	7.8 (5.1–10.5)	49.5 (33.0–66.0)	
		Forwards	16 (7.2)	7.3 (3.7–10.8)	39.8 (22.8–56.7)	0.095 †
		Three-quarters	16 (7.2)	8.3 (4.2–12.4)	59.2 (29.5–88.9)	
Neurological (CNS)			20 (9.0)	4.8 (2.7–7.0)	22.1 (14.4–29.7)	
		Forwards	11 (5.0)	5.0 (2.0–7.9)	19.8 (7.8–31.8)	0.228 †
		Three-quarters	9 (4.0)	4.7 (1.6–7.7)	24.8 (13.3–36.4)	
Skin			13 (5.9)	3.1 (1.4–4.8)	11.6 (0.7–22.6)	
		Forwards	5 (2.2)	2.2 (0.2–0.4)	5.2 (0.4–9.9)	0.999 †
		Three-quarters	8 (3.6)	4.1 (1.2–7.0)	15.7 (3.1–34.6)	
Tendinous			10 (4.5)	2.4 (0.9–3.9)	18.3 (1.3–35.2)	
		Forwards	7 (3.1)	3.2 (0.8–5.5)	20.0 (5.9–45.9)	0.062 †
		Three-quarters	3 (1.3)	1.5 (0.2–3.3)	14.3 (15.6–44.2)	
Neurological (PNS)			3 (1.3)	0.7 (0.1–1.5)	11.3 (25.2–47.9)	
		Forwards	3 (1.3)	1.3 (0.1–2.9)	11.3 (25.2–47.9)	*-*
		Three-quarters				
Other			3 (1.3)	0.7 (0.1–1.5)	16.0 (16.5–48.5)	
		Forwards	1 (0.4)	0.4 (0.4–1.3)	14.0	0.126 †
		Three-quarters	2 (0.9)	1.0 (0.4–2.4)	17.0 (8.1–18.1)	

CI = confidence intervals. * Kruskal-Wallis test was used. † Wilcoxon-Mann-Whitney test was used.

**Table 5:** Rate and seriousness of all injuries distributed by type of injury.

Injury	*p*-Value	Position	n (%)	Rate (95% CI:)	Seriousness (95% CI:)	*p*-Value
Concussion	<0.001 *		20 (9.0)	4.8 (2.7–7.0)	22.1 (14.4–29.7)	
		Forwards	11 (5.0)	5.0 (2.0–7.9)	19.8 (7.8–31.8)	0.380 †
		Three-quarters	9 (4.0)	4.7 (1.6–7.7)	24.8 (13.3–36.4)	
Spinal cord compression			2 (0.9)	0.4 (0.1–1.1)	3.0 (2.5–4.1)	
		Forwards	2 (0.9)	0.9 (0.3–2.1)	3.0 (2.5–4.1)	-
		Three-quarters				
Fracture			19 (8.6)	4.6 (2.5–6.7)	58.0 (39.8–76.1)	
		Forwards	12 (5.4)	5.4 (2.3–8.5)	50.1 (31.0–69.2)	0.190 †
		Three-quarters	7 (3.1)	3.6 (0.9–6.3)	71.4 (27.3–115.5)	
Other bone injuries			7 (3.1)	1.7 (0.4–2.9)	54.4 (9.7–118.5)	
		Forwards	2 (0.9)	0.9 (0.3–2.1)	11.0 (9.8–61.8)	0.121 †
		Three-quarters	5 (2.2)	2.6 (0.3–4.9)	71.8 (23.4–167.0)	
Dislocation/subluxation			13 (5.9)	3.1 (1.5–4.8)	95.0 (32.3–157.6)	
		Forwards	7 (3.1)	3.2 (0.8–5.5)	95.7 (2.4–188.9)	0.808 †
		Three-quarters	6 (2.7)	3.1 (0.6–5.6)	94.1 (28.3–216.6)	
Sprain/ligament injury			72 (32.7)	17.5 (13.5–21.5)	43.4 (30.0–56.8)	
		Forwards	42 (19.0)	19.2 (13.4–24.9)	35.5 (20.1–50.8)	0.299 †
		Three-quarters	30 (13.6)	15.6 (10.1–21.2)	54.5 (29.8–79.1)	
Meniscus, cartilage or disk injury			1 (0.4)	0.2 (0.2–0.7)	48.0	
		Forwards				-
		Three-quarters	1 (0.4)	0.5 (0.5–1.5)	48.0	
Muscle rupture, overload, tear or cramps			46 (20.9)	11.2 (8.0–14.4)	25.3 (16.2–34.3)	
		Forwards	34 (15.4)	15.5 (10.3–20.7)	25.4 (14.4–36.3)	0.373 †
		Three-quarters	12 (5.4)	6.2 (2.7–9.8)	25.0 (6.8–43.1)	
Tendinopathies, tendon rupture or bursitis			10 (4.5)	2.4 (0.9–3.9)	18.3 (1.3–35.2)	
		Forwards	7 (3.1)	3.2 (0.8–5.5)	20.0 (5.9–45.9)	0.909 †
		Three-quarters	3 (1.3)	1.5 (0.2–3.3)	14.3 (13.6–44.2)	
Bruises, hits or contusions			14 (6.3)	3.4 (1.6–5.2)	16.4 (6.6–26.2)	
		Forwards	3 (1.3)	1.3 (0.1–2.9)	5.6 (0.5–10.8)	0.359 †
		Three-quarters	11 (5.0)	5.7 (2.3–9.1)	19.3 (7.1–31.5)	
Laceration, cut or wound			13 (5.9)	3.1 (1.4–4.8)	11.6 (0.7–22.6)	
		Forwards	5 (2.2)	2.2 (0.2–4.2)	5.2 (0.4–9.9)	0.233 †
		Three-quarters	8 (3.6)	4.1 (1.2–7.0)	15.7 (3.1–34.6)	
Peripheral nerve injury			1 (0.4)	0.2 (0.2–0.7)	28.0	
		Forwards	1 (0.4)	0.4 (0.4–1.3)	28.0	-
		Three-quarters				
Tooth injury			2 (0.9)	0.4 (0.1–1.1)	9.0 (5.5–72.5)	
		Forwards	1 (0.4)	0.4 (0.4–1.3)	14.0	-
		Three-quarters	1 (0.4)	0.5 (0.5–1.5)	4.0	

CI = confidence intervals. * Kruskal-Wallis test was used. † Wilcoxon-Mann-Whitney test was used.

**Table 6:** Events associated with match injuries.

Event	*p*-Value	Position	n (%)	Rate (95% CI:)	Seriousness (95% CI:)	*p*-Value
Non-contact	<0.001 *		47 (21.3)	11.4 (8.2–14.7)	30.3 (18.6–42.1)	
		Forwards	28 (12.7)	12.8 (8.0–17.5)	19.4 (12.0–26.8)	0.180 †
		Three-quarters	19 (8.6)	9.9 (5.4–14.3)	46.5 (19.7–73.2)	
Being tackled			46 (20.9)	11.2 (8.0–14.4)	56.8 (35.8–77.8)	
		Forwards	24 (10.9)	10.9 (6.6–15.3)	54.3 (23.8–84.8)	0.848 †
		Three-quarters	22 (10.0)	11.5 (6.7–16.2)	59.4 (28.1–90.7)	
Tackling			53 (24.0)	12.9 (9.4–16.3)	40.1 (24.3–56.0)	
		Forwards	26 (11.8)	11.8 (7.3–16.4)	31.0 (12.1–49.9)	0.064 †
		Three-quarters	27 (12.2)	14.1 (8.8–19.4)	48.9 (22.9–75.5)	
Maul			2 (0.9)	0.4 (0.1–1.1)	52.5 (47.1–262.1)	
		Forwards	2 (0.9)	0.9 (0.3–2.1)	52.5 (47.1–262.1)	-
		Three-quarters				
Ruck			25 (11.3)	6.1 (3.7–8.4)	27.2 (11.8–42.6)	
		Forwards	17 (7.7)	7.7 (4.0–11.4)	27.5 (5.0–50.1)	0.105 †
		Three-quarters	8 (3.6)	4.1 (1.2–7.0)	26.5 (10.1–42.8)	
Touche			4 (1.8)	0.9 (0.0–1.9)	25.2 (11.2–39.2)	
		Forwards	4 (1.8)	1.8 (0.0–3.6)	25.2 (11.2–39.2)	-
		Three-quarters				
Scrum			9 (4.0)	2.2 (0.7–3.6)	31.6 (8.1–55.2)	
		Forwards	9 (4.0)	4.1 (1.4–6.8)	31.6 (8.1–55.2)	-
		Three-quarters				
Colllslon			25 (11.3)	6.1 (3.7–8.4)	27.0 (15.1–38.9)	
		Forwards	12 (5.4)	5.4 (2.3–8.5)	36.0 (14.3–57.6)	0.252 †
		Three-quarters	13 (5.9)	6.8 (3.1–10.4)	18.7 (5.9–31.6)	
Other			9 (4.0)	2.2 (0.7–3.6)	10.8 (5.9–15.8)	
		Forwards	5 (2.2)	2.2 (0.2–4.2)	10.2 (1.8–18.5)	0.621 †
		Three-quarters	4 (1.8)	2.0 (0.0–4.1)	11.7 (0.7–22.7)	

CI = confidence intervals. * Kruskal-Wallis test was used. † Wilcoxon-Mann-Whitney test was used.

**Table 7:** Injuries by match quarter.

Match Quarter	*p*-Value	Position	n (%)	Rate (95% CI:)	Seriousness (95% CI:)	*p*-Value
First quarter	<0.029 *		32 (14.5)	7.8 (5.1–10.5)	38.0 (22.8–53.2)	
		Forwards	13 (5.9)	5.9 (2.7–9.1)	23.4 (13.8–33.1)	0.399 †
		Three-quarters	19 (8.6)	9.9 (5.4–14.3)	48.0 (23.3–72.7)	
Second quarter			49 (22.2)	11.9 (8.6–15.2)	52.8 (31.7–73.8)	
		Forwards	26 (11.8)	11.8 (7.2–16.4)	43.4 (18.1–68.7)	0.976 †
		Three-quarters	23 (10.4)	12.0 (7.1–16.9)	63.3 (27.0–99.6)	
Third quarter			77 (35.0)	18.7 (14.6–22.9)	27.2 (20.2–34.3)	
		Forwards	52 (23.6)	23.7 (17.3–30.1)	24.7 (18.0–31.4)	0.557 †
		Three-quarters	25 (11.3)	13.0 (7.9–18.1)	32.6 (15.3–49.9)	
Fourth quarter			62 (28.1)	15.1 (11.3–18.8)	35.6 (22.6–48.6)	
		Forwards	36 (16.3)	16.4 (11.3–21.7)	38.2 (18.6–58.8)	0.723 †
		Three-quarters	26 (11.8)	13.5 (8.4–18.7)	31.9 (15.5–48.4)	

CI = confidence intervals. * Kruskal-Wallis test was used. † Wilcoxon-Mann-Whitney test was used.

**Table 8:** New vs. recurrent injuries.

Position	Injury	n (%)	Rate (95% CI:)	Seriousness (95% CI:)	*p*-Value
All		220	53.6 (46.7–60.5)	36.8 (30.1–43.6)	0.088 †
	New	167 (75.9)	40.7 (34.6–46.7)	40.8 (32.3–49.3)	
	Recurrent	53 (24.0)	12.9 (9.4–16.3)	24.3 (16.7–31.8)	
Forwards		127 (57.7)	58.0 (48.2–67.8)	32.2 (20.3–40.2)	0.300 †
	New	98 (44.5)	44.8 (36.1–53.4)	35.2 (25.3–45.2)	
	Recurrent	29 (13.1)	13.2 (8.4–18.0)	22.1 (13.5–30.7)	
Three-quarters		93 (42.2)	48.6 (38.9–58.2)	40.5 (30.6–50.4)	0.288 †
	New	69 (31.3)	36.0 (27.7–44.4)	48.8 (33.7–63.9)	
	Recurrent	24 (10.9)	12.5 (7.5–17.5)	26.9 (13.2–40.6)	

CI = confidence intervals. † Wilcoxon-Mann-Whitney test was used.

## Data Availability

The datasets used and/or analysed during the current study are available from the corresponding author on reasonable request.
